# Supplementary material for: The distinct translational landscapes of gram-negative Salmonella and gram-positive Listeria
Source: Nat Commun. 2023 Dec 9;14:8167. doi: 10.1038/s41467-023-43759-1 (PMC10710512; doi:10.1038/s41467-023-43759-1)
Supplement: Supplementary file 1 — Supplementary Information [file 41467_2023_43759_MOESM1_ESM.pdf]

Supplementary information:
The supplementary information contains all supplementary figures, tables and extended methods with associated references.

Figure S1

A

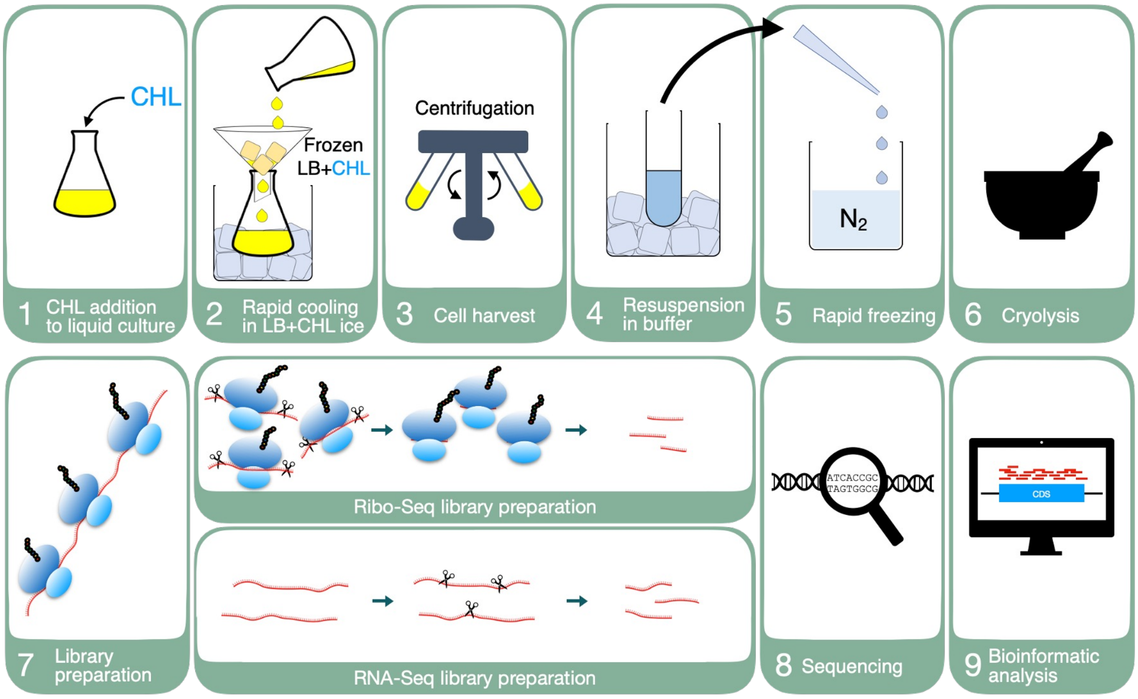

CHL
1 CHL addition to liquid culture
Frozen LB+CHL
2 Rapid cooling in LB+CHL ice
Centrifugation
3 Cell harvest
4 Resuspension in buffer
N2
5 Rapid freezing
6 Cryolysis
7 Library preparation
Ribo-Seq library preparation
RNA-Seq library preparation
ATCACCGC
TAGTGGCG
8 Sequencing
CDS
9 Bioinformatic analysis

B

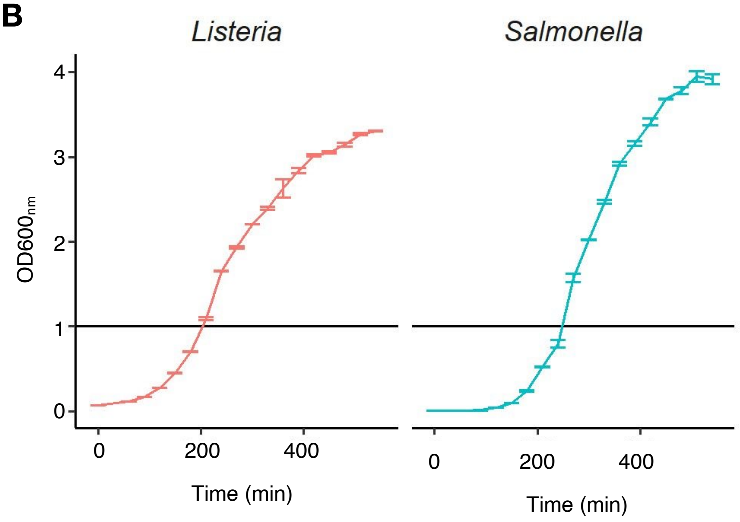

Listeria
Salmonella
OD600nm
0
1
2
3
4
0
200
400
Time (min)

Figure S1.
A. A schematic illustrating our revised RiboSeq and RNA sequencing method. Translation was arrested by treating cells with highly concentrated chloramphenicol immediately prior to harvesting cells by rapid cooling, followed by centrifugation and flash freezing in liquid nitrogen for cryolysis. Clarified supernatants were used to generate RiboSeq and RNASeq libraries. Figure created with BioRender.com.
B. Growth curves of Salmonella (left) and Listeria (right) in LB and BHI respectively. Optical density at $OD600_{nm}$ was measured every 30 mins. Results represent three independent experiments.

Figure S2

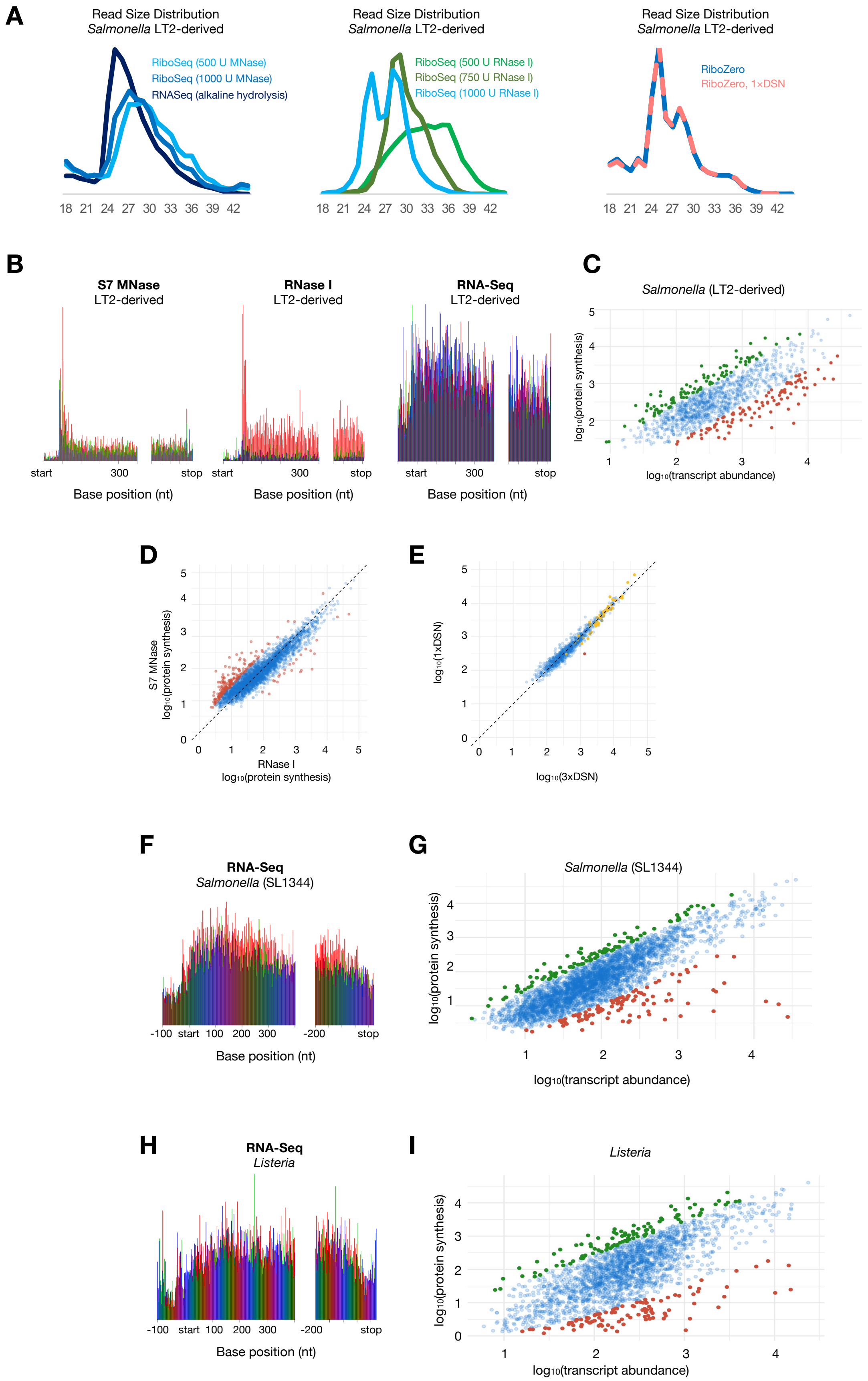
A
Read Size Distribution
Salmonella LT2-derived
RiboSeq (500 U MNase)
RiboSeq (1000 U MNase)
RNASeq (alkaline hydrolysis)
18 21 24 27 30 33 36 39 42
Read Size Distribution
Salmonella LT2-derived
RiboSeq (500 U RNase I)
RiboSeq (750 U RNase I)
RiboSeq (1000 U RNase I)
18 21 24 27 30 33 36 39 42
Read Size Distribution
Salmonella LT2-derived
RiboZero
RiboZero, 1×DSN
18 21 24 27 30 33 36 39 42
B
S7 MNase
LT2-derived
start 300 stop
Base position (nt)
RNase I
LT2-derived
start 300 stop
Base position (nt)
RNA-Seq
LT2-derived
start 300 stop
Base position (nt)
C
Salmonella (LT2-derived)
log10(protein synthesis)
log10(transcript abundance)
D
S7 MNase
log10(protein synthesis)
RNase I
log10(protein synthesis)
E
log10(1xDSN)
log10(3xDSN)
F
RNA-Seq
Salmonella (SL1344)
-100 start 100 200 300 -200 stop
Base position (nt)
G
Salmonella (SL1344)
log10(protein synthesis)
log10(transcript abundance)
H
RNA-Seq
Listeria
-100 start 100 200 300 -200 stop
Base position (nt)
I
Listeria
log10(protein synthesis)
log10(transcript abundance)

Figure S2.

A. Read length distribution of RiboSeq libraries from *Salmonella* generated with S7 MNase (500 U or 1000 U) and corresponding RNAseq library (left) or generated with RNase **I** (500 U, 750 U or 1000 U) (middle). The length distribution of RiboSeq libraries from *Salmonella* generated with RNase **I** (700 U) treated with RiboZero or with RiboZero in combination with DSN (right).

B. Metagene translatome and metagene transcriptome plots generated by aligning all open reading frames of LT2-derived *Salmonella* using the start and stop codons as anchors. RiboSeq libraries were generated with S7 MNase (1000 U, left), RNase **I** (700 U, middle). Reads that map to codon positions 1, 2 and 3 are coloured in red, green and blue, respectively. The plot was produced with the R software package riboSeqR[56].

C. Scatterplot showing the relationship between protein synthesis (i.e. RPF abundance) and transcript abundance (i.e. mRNA abundance) for all transcribed and translated *Salmonella* genes in the RNAse-I-treated LT2-derived *Salmonella*.

D. Comparison of normalised RPF count in LT2-derived *Salmonella* libraries treated with RNase **I** (700 U) or S7 MNase (1000 U). The read counts where the difference is greater than three-fold are highlighted in red. Genes with read counts lower than 50 in both libraries were rejected.

E. Comparison of normalised RPF count in *Listeria* libraries treated 1× or 3× with DSN. The read count where the difference is greater than three-fold is highlighted in red. Ribosomal genes are charted in yellow.

F. Metagene transcriptome plot of *Salmonella* SL1344 generated by aligning all coding sequences using their start and stop codons as anchors.

G. Scatterplot showing the relationship between total protein synthesis (i.e. RPF abundance) and transcript abundance (i.e. mRNA abundance) for all transcribed and translated genes of *Salmonella* SL1344.

H. Metagene transcriptome plot of the pathogenic *Listeria* 10403S generated by aligning all coding sequences using their start and stop codons as anchors. **I**. Scatterplot showing the relationship between protein synthesis (i.e. RPF abundance) and transcript abundance (i.e. mRNA abundance) for all transcribed and translated genes of Listeria 10403S.

Figure S3

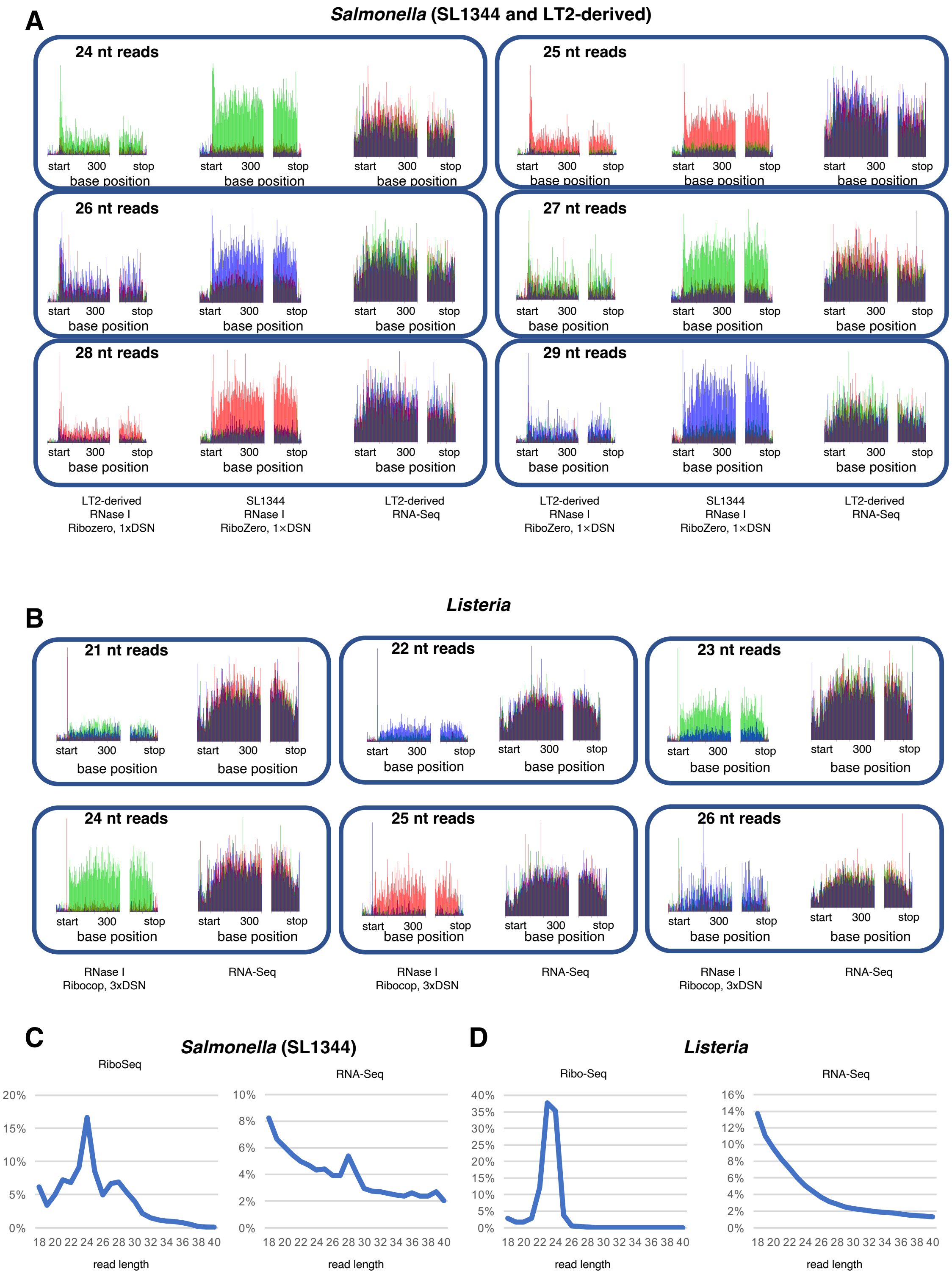
A
Salmonella (SL1344 and LT2-derived)
24 nt reads
25 nt reads
26 nt reads
27 nt reads
28 nt reads
29 nt reads
start 300 stop
base position
LT2-derived RNase I Ribozero, 1xDSN
SL1344 RNase I RiboZero, 1×DSN
LT2-derived RNA-Seq
LT2-derived RNase I RiboZero, 1×DSN
SL1344 RNase I RiboZero, 1×DSN
LT2-derived RNA-Seq
B
Listeria
21 nt reads
22 nt reads
23 nt reads
24 nt reads
25 nt reads
26 nt reads
RNase I Ribocop, 3xDSN
RNA-Seq
C
Salmonella (SL1344)
RiboSeq
RNA-Seq
D
Listeria
Ribo-Seq
RNA-Seq
20% 15% 10% 5% 0%
10% 8% 6% 4% 2% 0%
40% 35% 30% 25% 20% 15% 10% 5% 0%
16% 14% 12% 10% 8% 6% 4% 2% 0%
18 20 22 24 26 28 30 32 34 36 38 40
read length

Figure S3.
A. RiboSeq and parallel RNASeq generated from LT2-derived *Salmonella* strain or pathogenic SL1344 across different RPF sizes. Red, green and blue bars indicate 5′ positions of reads mapping to codon positions 1, 2 and 3, respectively.
B. RiboSeq and parallel RNASeq generated from *Listeria* strain 10403S across different RPF sizes. Red, green and blue bars indicate 5′ positions of reads mapping to codon positions 1, 2 and 3, respectively.
C and D. Read size distribution of RiboSeq (left) and RNA-Seq (right) in *Salmonella* and *Listeria*.

Figure S4

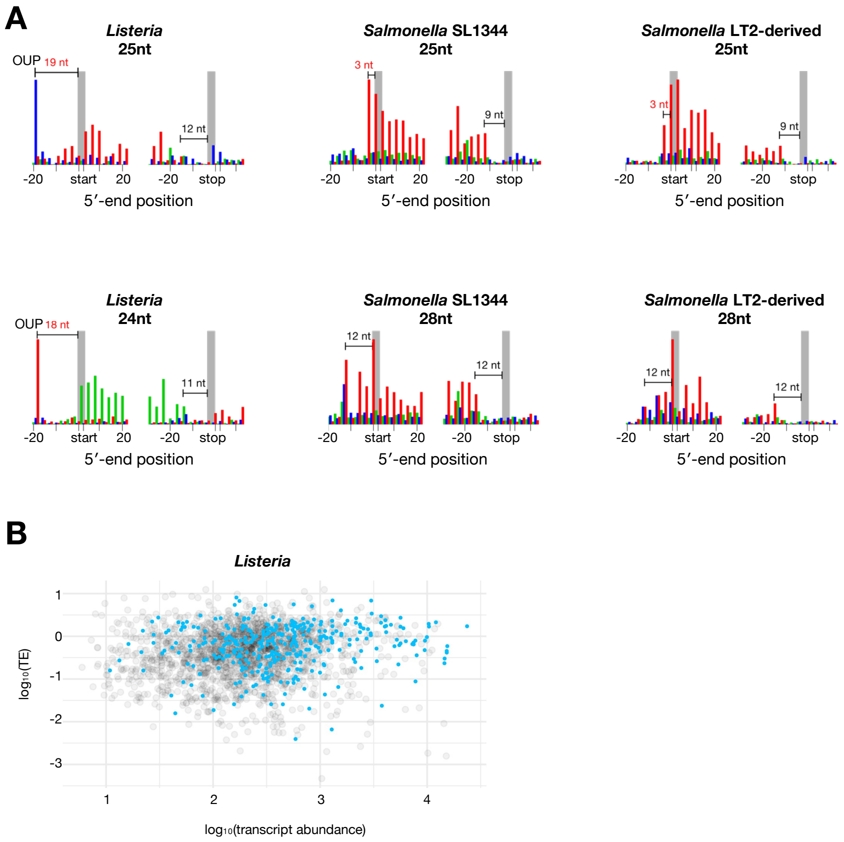
A
Listeria
25nt
OUP 19 nt
12 nt
-20
start
20
-20
stop
5′-end position
Salmonella SL1344
25nt
3 nt
9 nt
Salmonella LT2-derived
25nt
3 nt
9 nt
Listeria
24nt
OUP 18 nt
11 nt
Salmonella SL1344
28nt
12 nt
12 nt
Salmonella LT2-derived
28nt
12 nt
12 nt
B
Listeria
log10(TE)
log10(transcript abundance)
1
0
-1
-2
-3
1
2
3
4

Figure S4.
A. Detailed metagene translatome plots of *Listeria* 10403S, *Salmonella* SL1344 and LT2-derived *Salmonella*. Reads that map to codon positions 1, 2 and 3 are coloured in red, green and blue, respectively. P-site positions of initiating and terminating ribosomes are illustrated in grey and their distance to the 5′-most prominent peak is specified and highlighted in red if it deviates from the calculated 5′-end–P-site distance.
B. Scatterplot showing the relationship between protein synthesis (i.e. RPF abundance) and transcript abundance (i.e. mRNA abundance) of all transcribed and translated genes of *Listeria* 10403S. Genes containing the out-of-frame upstream peak (OUP) of at least ten reads are coloured in blue.

Figure S5

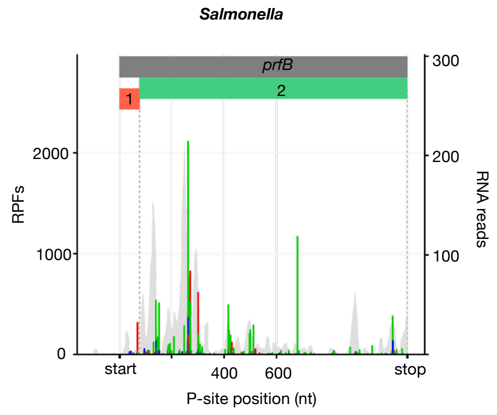
Salmonella
prfB
1
2
2000
1000
0
RPFs
300
200
100
RNA reads
start
400
600
stop
P-site position (nt)

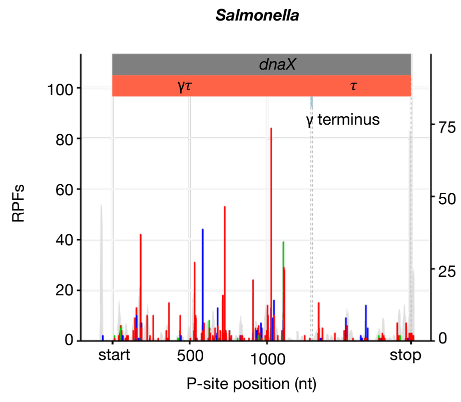
Salmonella
dnaX
γτ
τ
γ terminus
100
80
60
40
20
0
RPFs
75
50
25
0
start
500
1000
stop
P-site position (nt)

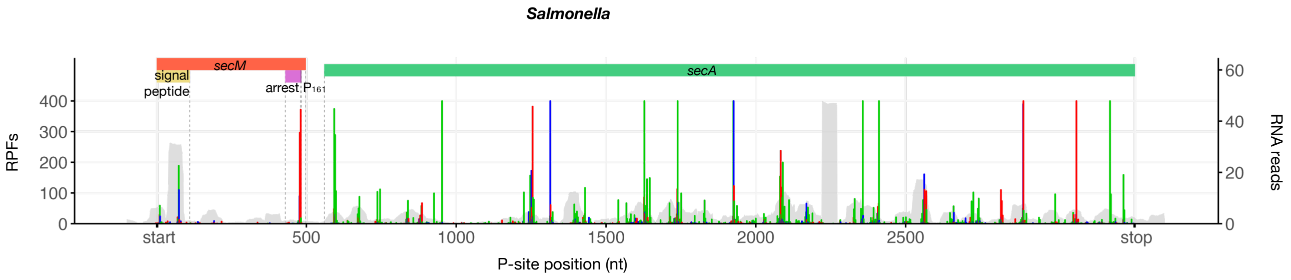
Salmonella
secM
secA
signal
peptide
arrest
P161
400
300
200
100
0
RPFs
60
40
20
0
RNA reads
start
500
1000
1500
2000
2500
stop
P-site position (nt)

Figure S5.
Visualisation of translation of *Salmonella prfB* (top), *dnaX* (middle), *secM* and *secA* (bottom). Red, green and blue bars indicate RiboSeq reads mapping to codon positions 1, 2 and 3, respectively. Grey shaded peaks show parallel RNASeq data.

Figure S6

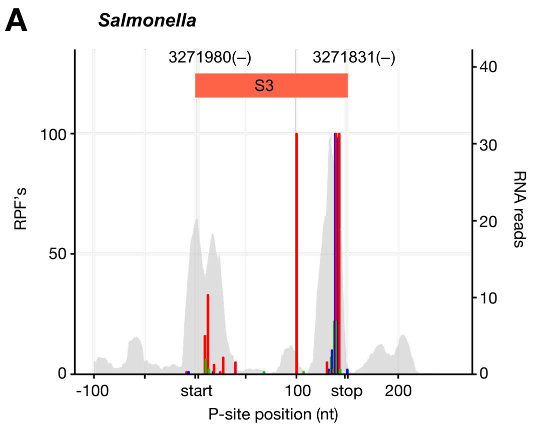
A
Salmonella
3271980(–)
3271831(–)
S3
RPF's
RNA reads
100
50
0
40
30
20
10
0
-100
start
100
stop
200
P-site position (nt)

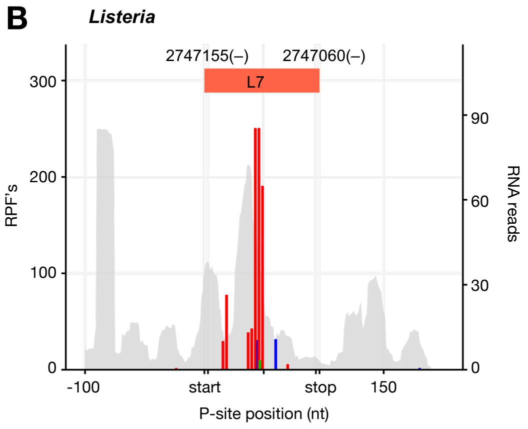
B
Listeria
2747155(–)
2747060(–)
L7
RPF's
RNA reads
300
200
100
0
90
60
30
0
-100
start
stop
150
P-site position (nt)

Figure S6.
Visualisation of translation of novel ORFs identified from the *Salmonella* RiboSeq data.
A. Candidate S3 identified in *Salmonella*.
B. Candidate L7 identified in *Listeria*.
Red, green and blue bars indicate RiboSeq reads mapping to codon positions 1, 2 and 3, respectively. Grey shaded peaks show parallel RNASeq data. The RPF read count is capped at 100 (S3) and 250 (L7) to display clearly peaks at positions with a lower read density.

Figure S7

A

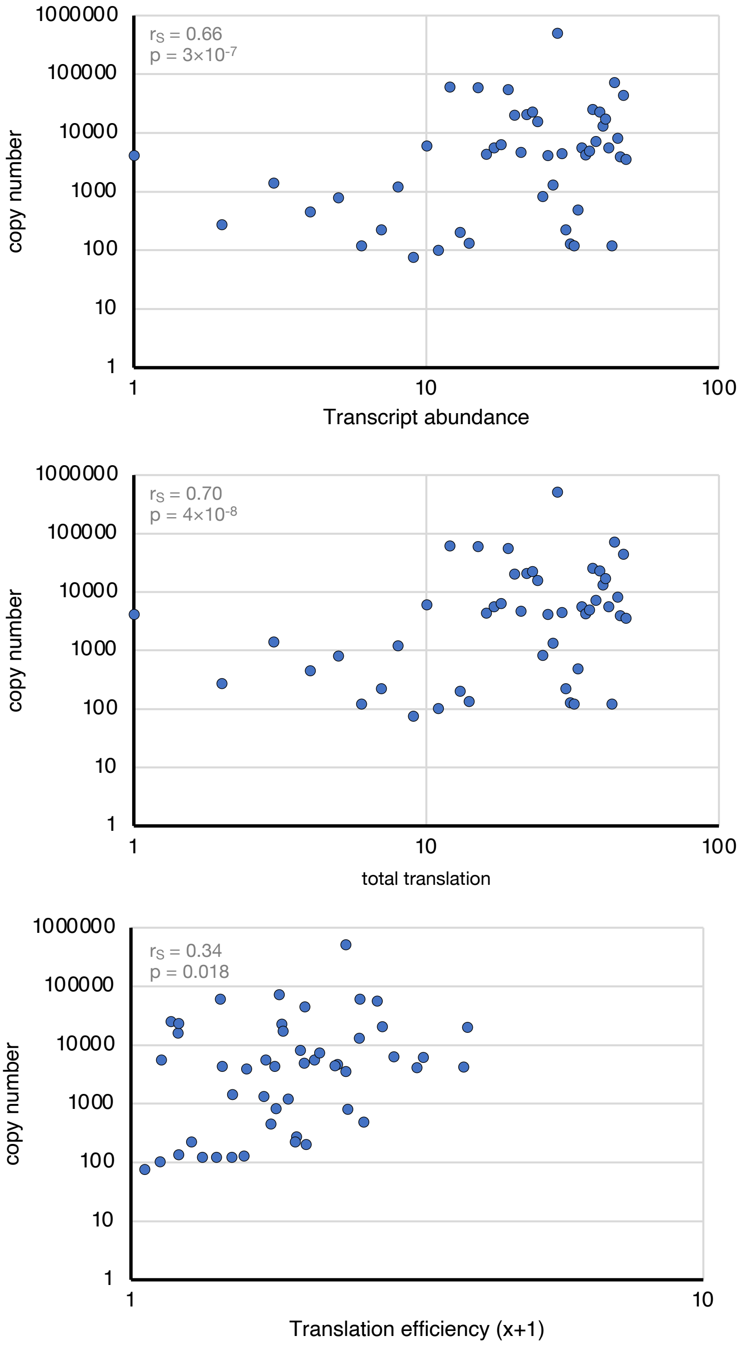

1000000
100000
10000
1000
100
10
1
copy number
r_S = 0.66
p = 3×10^{-7}
1
10
100
Transcript abundance
r_S = 0.70
p = 4×10^{-8}
total translation
r_S = 0.34
p = 0.018
Translation efficiency (x+1)

Figure S7.
Transcript abundance (top), protein synthesis (middle) and translation efficiency (bottom) plotted against the published copy number of proteins reported in the literature[98–112].

Figure S8

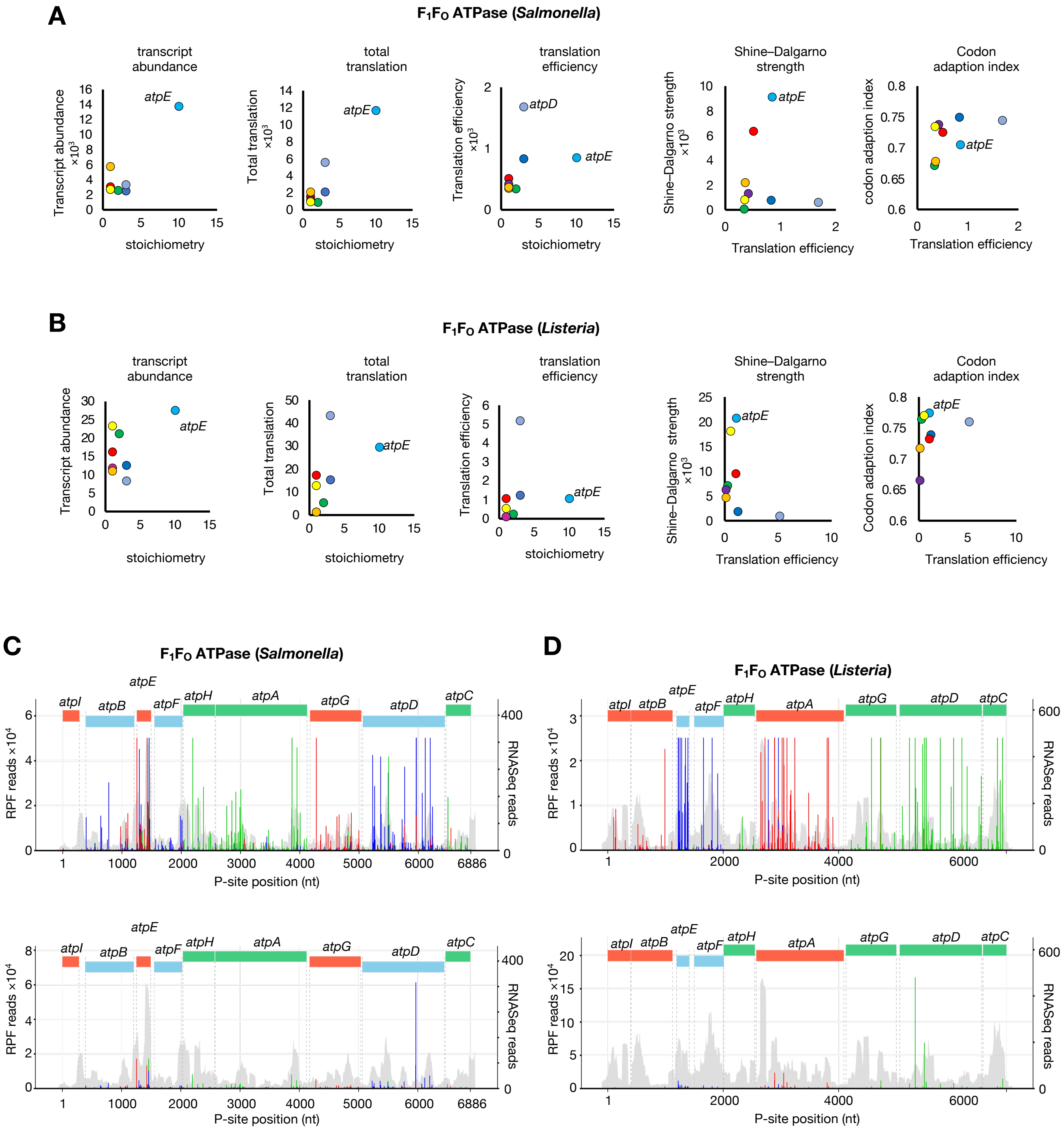

A
F1FO ATPase (Salmonella)
transcript abundance
total translation
translation efficiency
Shine–Dalgarno strength
Codon adaption index
Transcript abundance ×10³
Total translation ×10³
Translation efficiency ×10³
Shine–Dalgarno strength ×10³
codon adaption index
stoichiometry
Translation efficiency
atpE
atpD
B
F1FO ATPase (Listeria)
transcript abundance
total translation
translation efficiency
Shine–Dalgarno strength
Codon adaption index
Transcript abundance
Total translation
Translation efficiency
Shine–Dalgarno strength ×10³
Codon adaption index
stoichiometry
Translation efficiency
atpE
C
F1FO ATPase (Salmonella)
D
F1FO ATPase (Listeria)
atpI
atpB
atpE
atpF
atpH
atpA
atpG
atpD
atpC
RPF reads ×10⁴
RNASeq reads
P-site position (nt)

Figure S8.

A and B. Relationship between stoichiometry of F_1F_0 ATPase complex subunits and transcript abundance, protein synthesis or translation efficiency of *Salmonella* (A) and *Listeria* (B). The panels on the right show the relationship of translation efficiency with SD strength and codon adaptation index, respectively. Data points corresponding to the *atpE* gene (blue) were outliers and included in the generation of these charts (but excluded from the equivalent charts in Figure 2).

C and D. Visualisation of the translation of *Salmonella* (C) and *Listeria* (D) *atp* operon, which contains the genes encoding components of the F_1F_o-ATPase complex. Red, green and blue bars indicate RiboSeq reads mapping to frames 1, 2 and 3, respectively. Grey shaded peaks show parallel RNASeq data. RiboSeq axis adjusted to enable visualisation of less translated ORFs (top) and unadjusted (bottom).

Figure S9

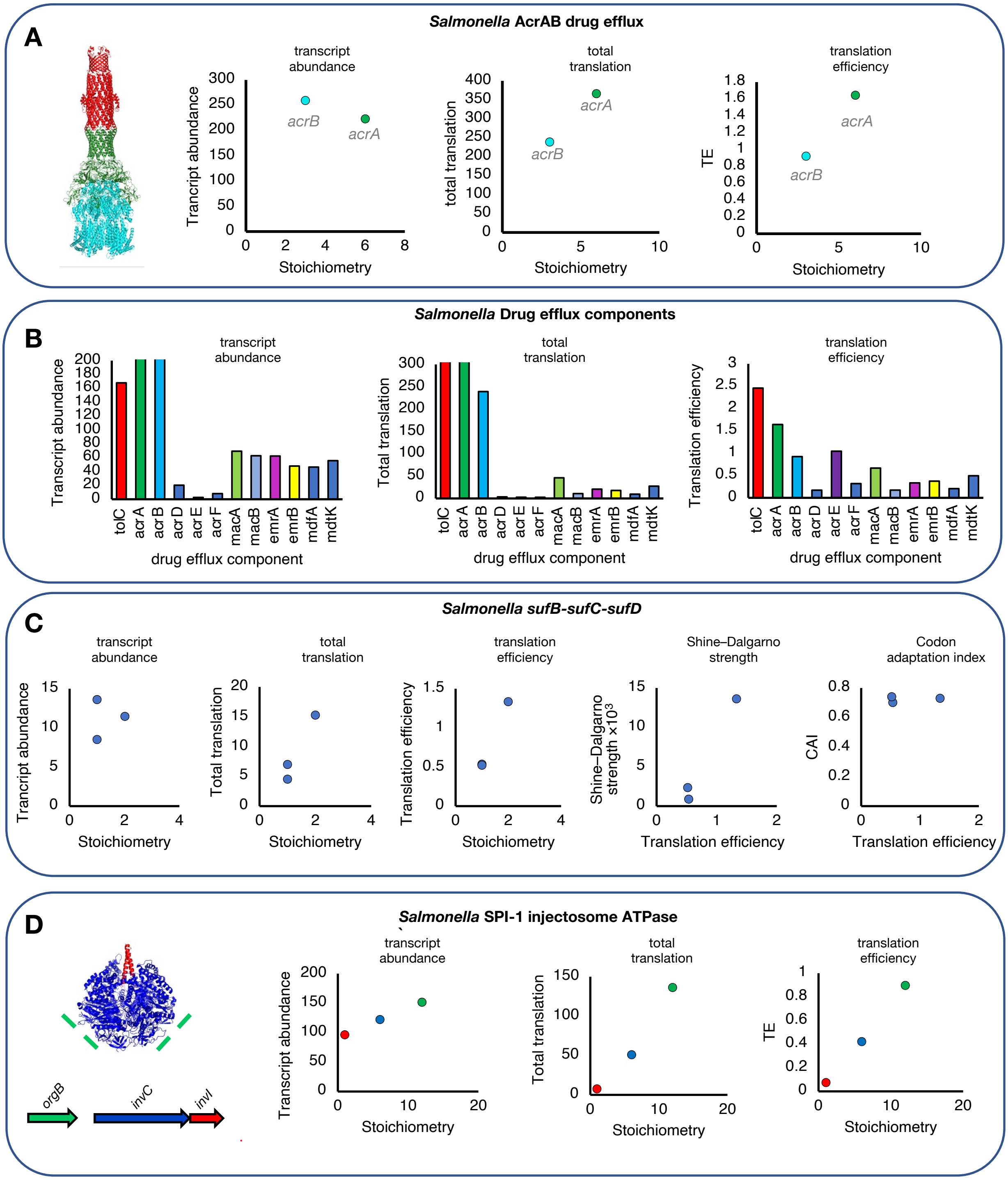

A
Salmonella AcrAB drug efflux
transcript abundance
Trancript abundance
Stoichiometry
acrB
acrA
total translation
total translation
translation efficiency
TE
B
Salmonella Drug efflux components
Transcript abundance
Total translation
Translation efficiency
tolC
acrA
acrB
acrD
acrE
acrF
macA
macB
emrA
emrB
mdfA
mdtK
drug efflux component
C
Salmonella sufB-sufC-sufD
Shine–Dalgarno strength
Shine–Dalgarno strength ×10³
Codon adaptation index
CAI
Translation efficiency
D
Salmonella SPI-1 injectosome ATPase
orgB
invC
invI

Figure S9.
A. Structure of the bacterial AcrAB-TolC drug efflux pump complex[113]. Charts showing the correlation between the stoichiometry of the *Salmonella* AcrA/AcrB efflux pump components and the transcript abundance, total protein synthesis or translation efficiency of genes that encode them.
B. Transcript abundance, total protein synthesis and translation efficiency of a representative range of bacterial drug efflux pump components of *Salmonella*.
C. Charts showing the correlation between the stoichiometry of the *Salmonella* SufB/SufC/SufD components and the transcript abundance, protein synthesis or translation efficiency of genes that encode them (left). The relationship of SD strength or codon adaptation index (CAI) with translation efficiency is charted on the right.
D. Structural model of the *Salmonella* SPI-1 ATPase complex. Charts showing the correlation between the stoichiometry of the *Salmonella* OrgB (green), InvC (blue) and InvI (red) ATPase components and the transcript abundance, protein synthesis or translation efficiency of genes that encode them.

Figure S10
LISTERIA

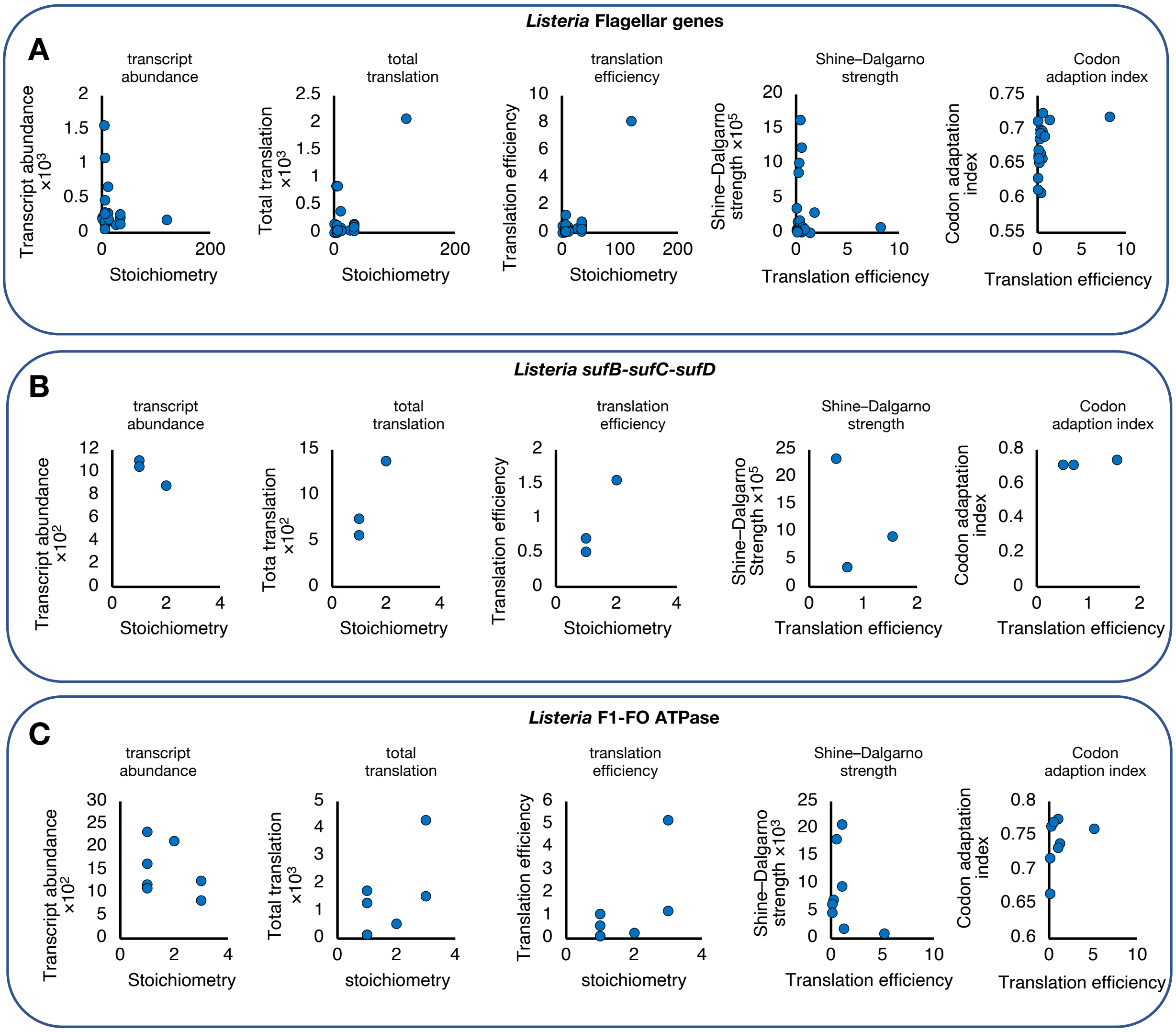

Figure S10.

A–C. Charts showing the correlation between the stoichiometry of the *Listeria* flagella (A), SufB–D (B) or F_1F_O-ATPase (C) components and the transcript abundance, protein synthesis or translation efficiency of genes that encode them. The relationship of SD strength or codon adaptation index (CAI) with translation efficiency is charted on the right.

Figure S11

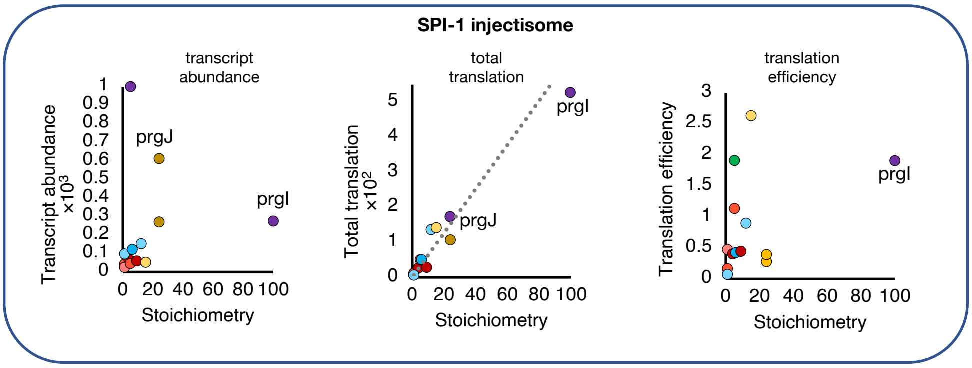
SPI-1 injectisome
transcript abundance
Transcript abundance ×10³
1
0.9
0.8
0.7
0.6
0.5
0.4
0.3
0.2
0.1
0
prgJ
prgI
0 20 40 60 80 100
Stoichiometry
total translation
Total translation ×10²
5
4
3
2
1
0
prgI
prgJ
0 20 40 60 80 100
Stoichiometry
translation efficiency
Translation efficiency
3
2.5
2
1.5
1
0.5
0
prgI
0 20 40 60 80 100
Stoichiometry

Figure S11.

The relationship between stoichiometry of SPI-1 vT3SS structural genes and the corresponding transcript abundance, protein synthesis or translation efficiency. PrgJ and PrgI are indicated on the chart.

Figure S12

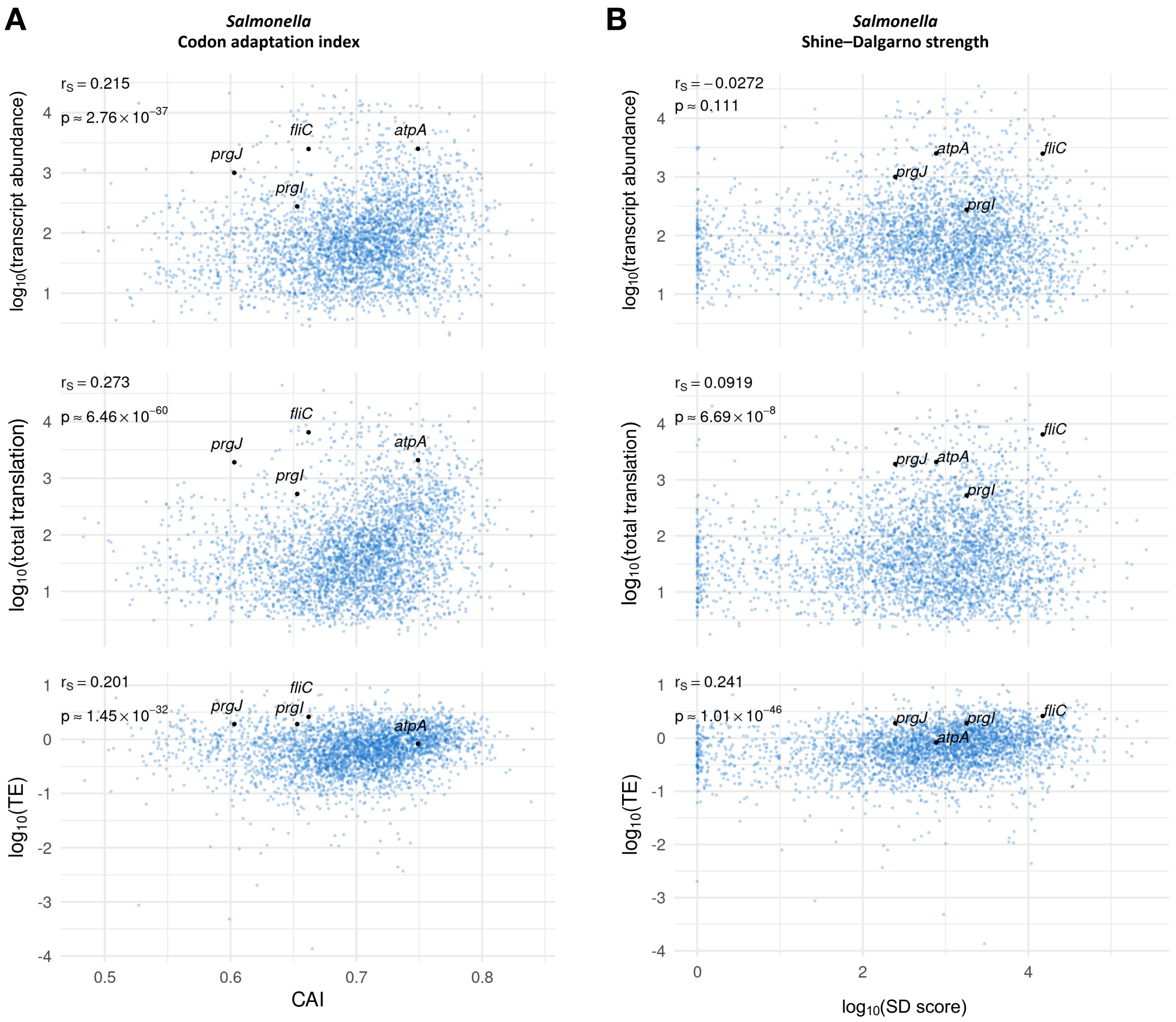
A
Salmonella
Codon adaptation index
B
Salmonella
Shine–Dalgarno strength
$r_S = 0.215$
$p \approx 2.76 \times 10^{-37}$
$r_S = 0.273$
$p \approx 6.46 \times 10^{-60}$
$r_S = 0.201$
$p \approx 1.45 \times 10^{-32}$
$r_S = -0.0272$
$p \approx 0.111$
$r_S = 0.0919$
$p \approx 6.69 \times 10^{-8}$
$r_S = 0.241$
$p \approx 1.01 \times 10^{-46}$
fliC
atpA
prgJ
prgI
$\log_{10}$(transcript abundance)
$\log_{10}$(total translation)
$\log_{10}$(TE)
CAI
$\log_{10}$(SD score)

Figure S12.
A and B. Scatterplots showing the relationship between the codon adaptation index (CAI; A) or SD strength (B) with the transcript abundance, protein synthesis and translation efficiency of ribosomal protein genes in *Salmonella*.

Figure S13

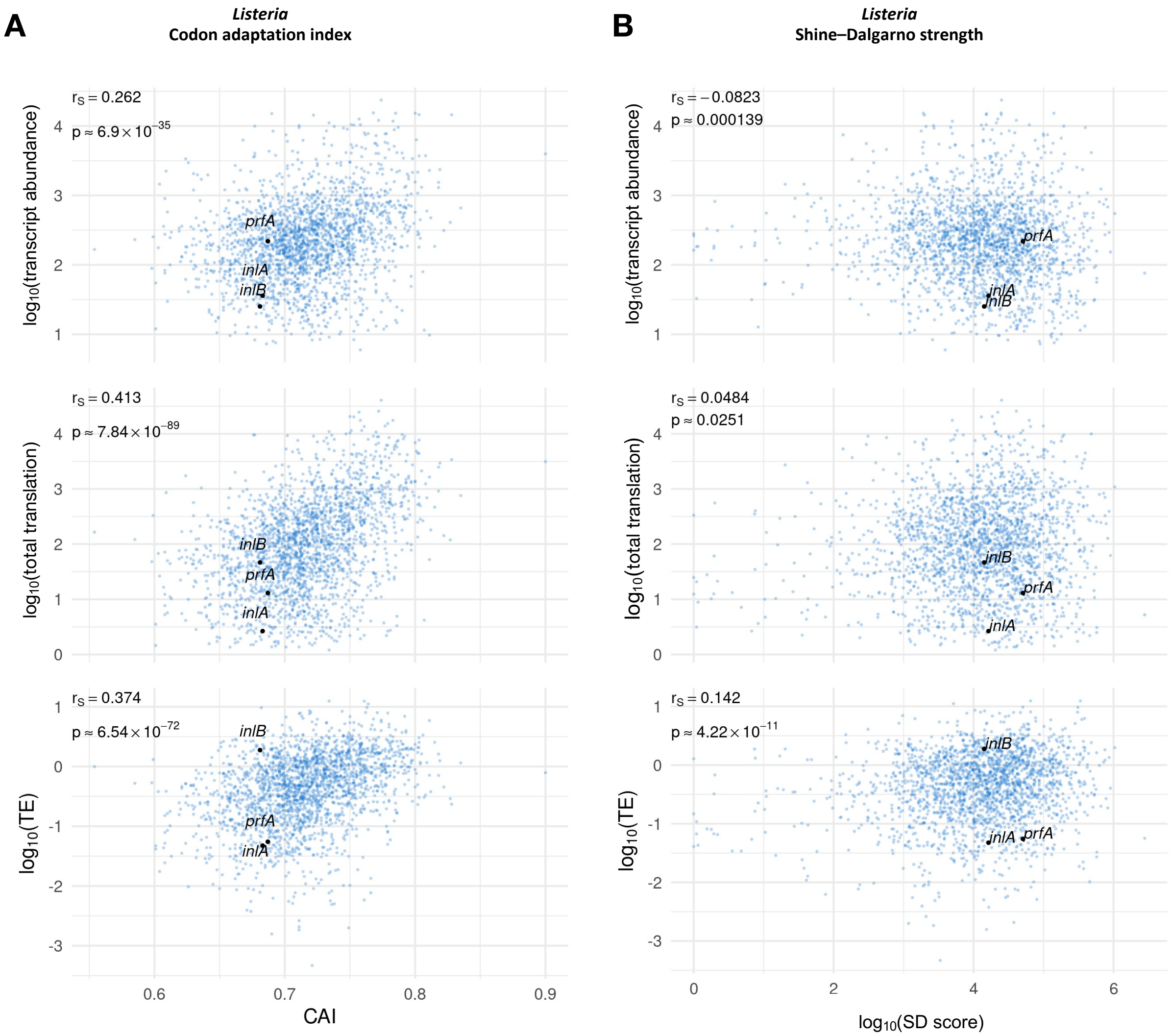
A
Listeria
Codon adaptation index
B
Listeria
Shine–Dalgarno strength
$r_S = 0.262$
$p \approx 6.9 \times 10^{-35}$
$r_S = -0.0823$
$p \approx 0.000139$
$r_S = 0.413$
$p \approx 7.84 \times 10^{-89}$
$r_S = 0.0484$
$p \approx 0.0251$
$r_S = 0.374$
$p \approx 6.54 \times 10^{-72}$
$r_S = 0.142$
$p \approx 4.22 \times 10^{-11}$
prfA
inlA
inlB
$\log_{10}$(transcript abundance)
$\log_{10}$(total translation)
$\log_{10}$(TE)
CAI
$\log_{10}$(SD score)

Figure S13.
A and B. Scatterplots showing the relationship between the codon adaptation index (CAI; A) or SD strength (B) with the transcript abundance, protein synthesis and translation efficiency of ribosomal protein genes in *Listeria*.

Figure S14 NEW

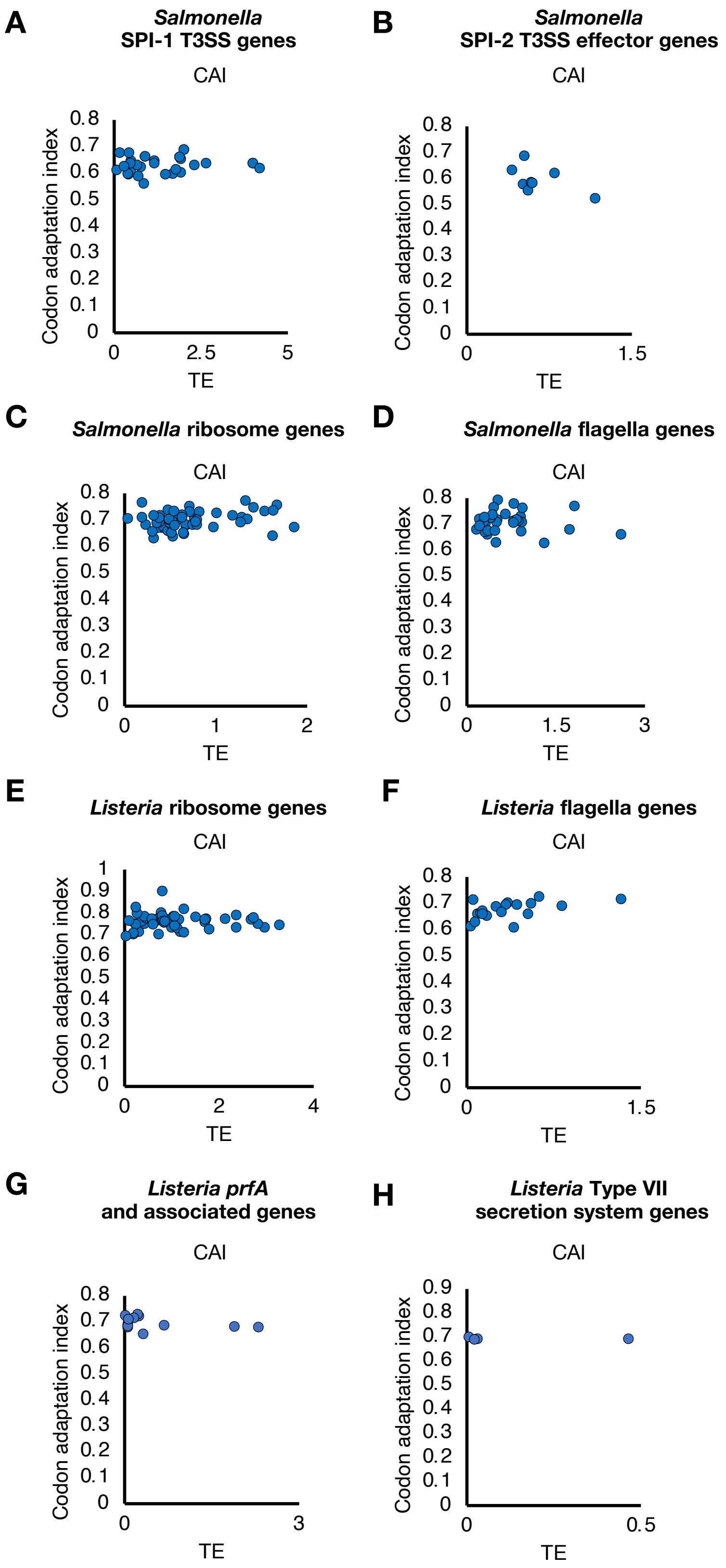

Figure S14.
Scatterplots showing the relationship between the codon adaptation index and translation efficiency for SPI-1 T3SS and SPI-2 T3SS genes (**A, B**); ribosomal protein and flagella genes for *Salmonella* and *Listeria*, respectively (**C–F**); and *prfA* and associated genes as well as Type VII secretion system genes in *Listeria* **(G, H)**.

Figure S15 NEW

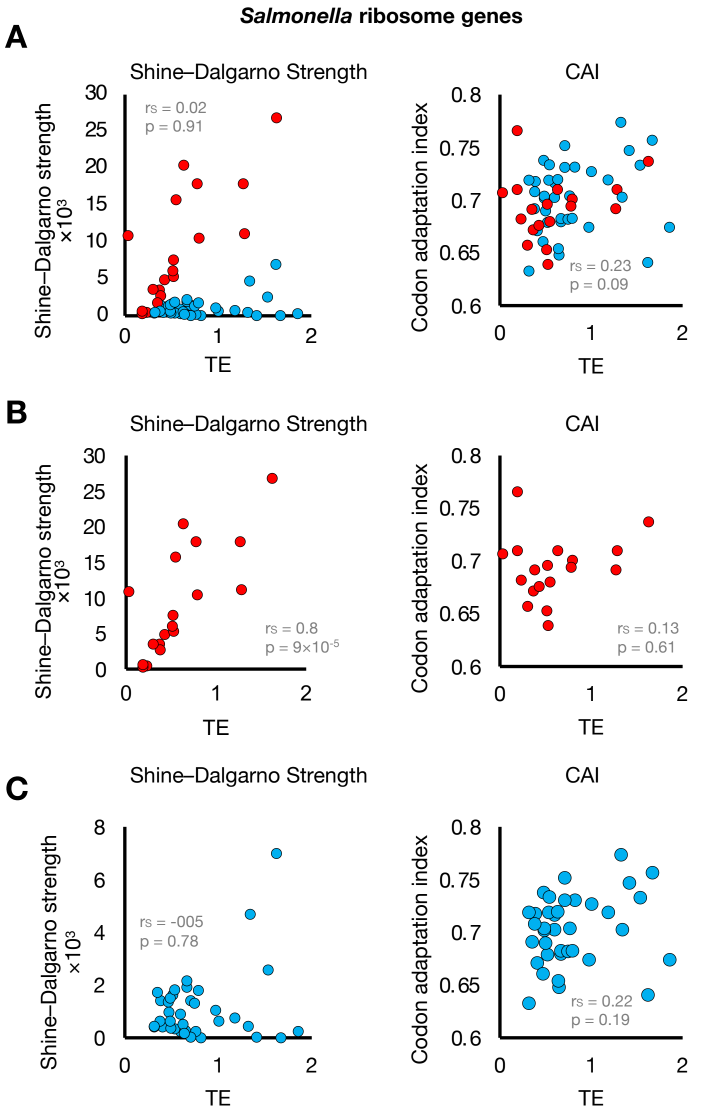

Figure S15 Scatterplot showing the relationship between the codon adaptation index and translation efficiency for all *Salmonella* ribosome genes, highlighting the two classes of genes (red or blue) (**A**) or the two classes of genes plotted separately (**B and C**). Spearman's correlation coefficient and the approximate p value have been calculated.

Figure S16 NEW

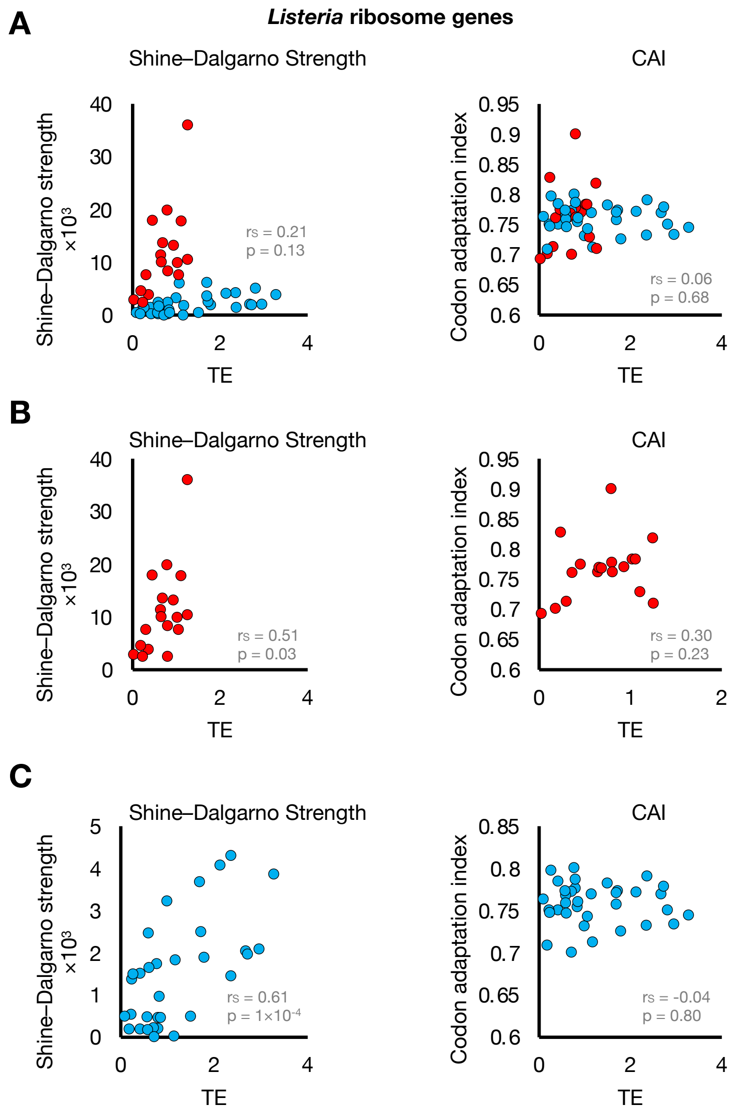

Figure S16
Scatterplot showing the relationship between the codon adaptation index with translation efficiency for all *Listeria* ribosome genes, highlighting the two classes of genes (red or blue) (**A**) or the two classes of genes plotted separately (**B and C**). Spearman's correlation coefficient and the approximate p value have been calculated.

Figure S17 NEW

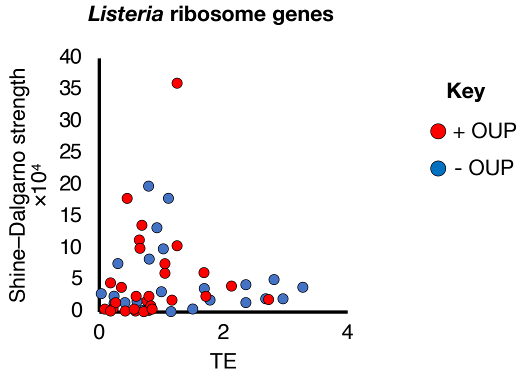

Figure S17
Scatterplot showing the relationship between Shine–Dalgarno strength and translation efficiency for *Listeria* ribosome genes highlighting OUP genes (red) and non-OUP genes (blue).

Figure S18

Salmonella

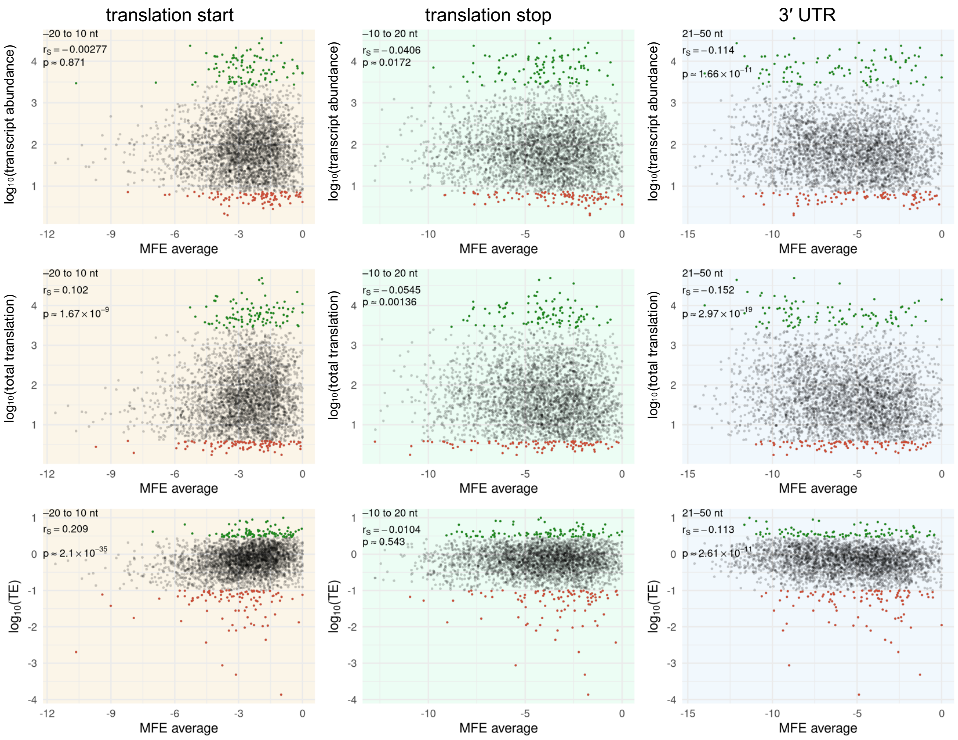

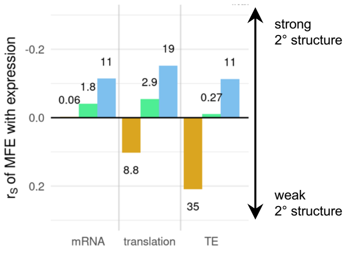

Figure S18.

Scatterplots showing the relationship of mRNA abundance, protein synthesis or translation efficiency with average MFE values for RNA secondary structure at the translation start site (left), translation stop site (middle) and in the 3′ UTR of all *Salmonella* genes. (See figure 4A.) Spearman's correlation coefficient and the approximate p value have been calculated. A summary of the Spearman's rank coefficient for each plot is given at the bottom.

Figure S19

Listeria

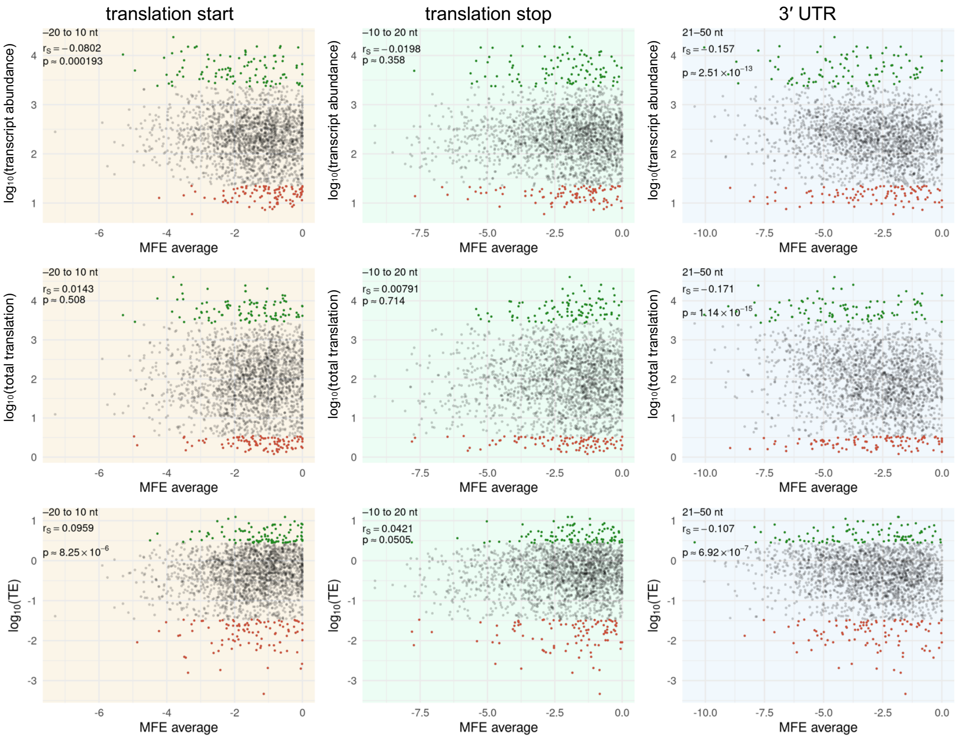

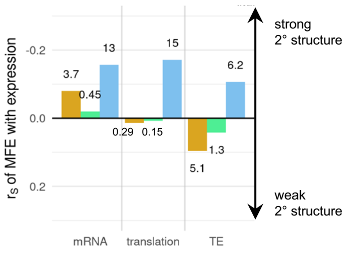

Figure S19.
Scatterplots showing the relationship between mRNA abundance, protein synthesis and translation efficiency and average MFE values for RNA secondary structure at the translation start site (left), translation stop site (middle) and in the 3′ UTR for all *Listeria* genes. (See figure 4B.) Spearman's correlation coefficient and the approximate p value have been calculated. A summary of the Spearman's rank coefficient for each plot is given at the bottom.

Figure S20

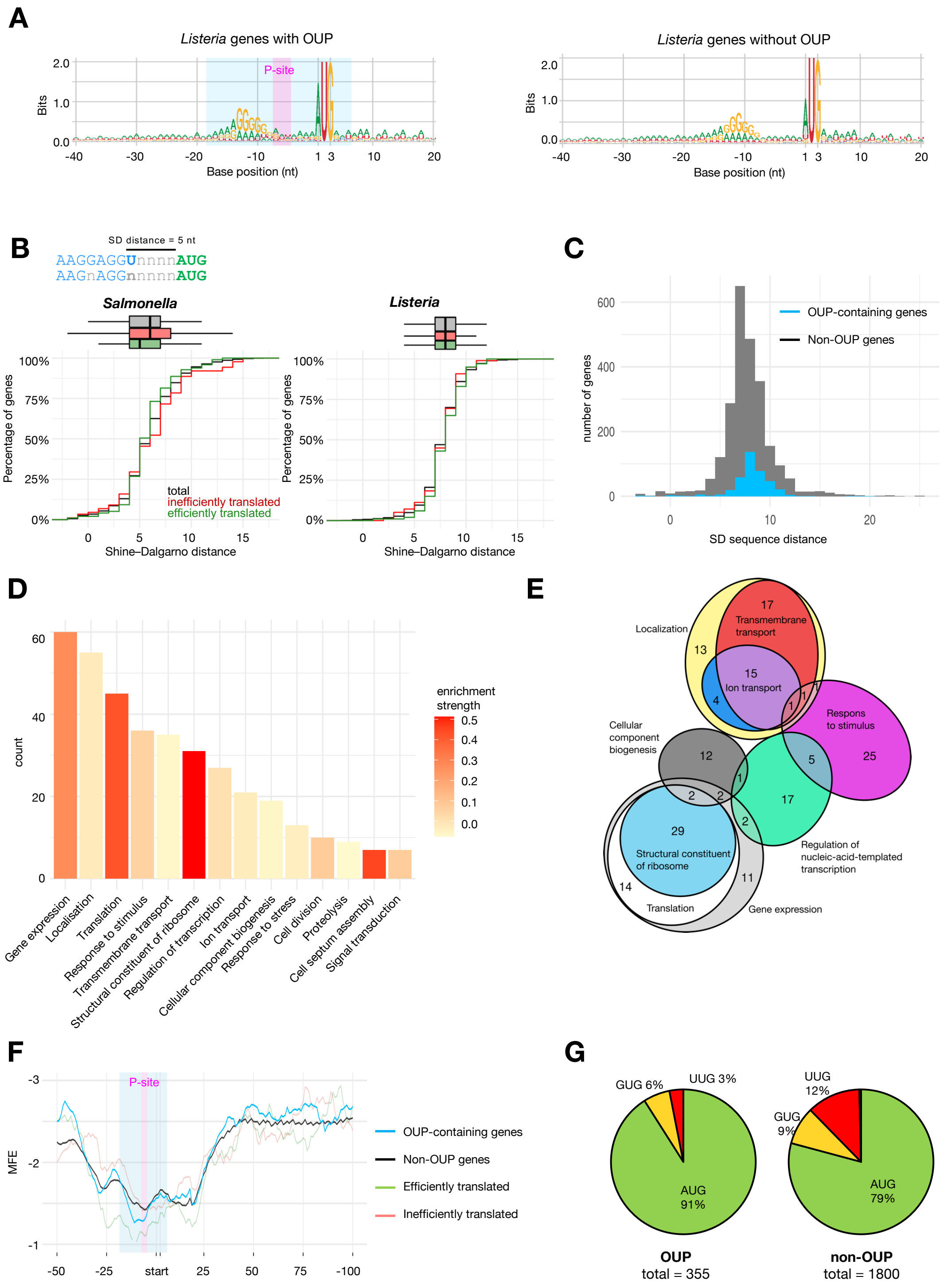

A
Listeria genes with OUP
P-site
Bits
Base position (nt)
Listeria genes without OUP
B
SD distance = 5 nt
AAGGAGGUnnnnnAUG
AAGnAGGnnnnnnAUG
Salmonella
Listeria
Percentage of genes
Shine–Dalgarno distance
total
inefficiently translated
efficiently translated
C
OUP-containing genes
Non-OUP genes
number of genes
SD sequence distance
D
count
enrichment strength
Gene expression
Localisation
Translation
Response to stimulus
Transmembrane transport
Structural constituent of ribosome
Regulation of transcription
Ion transport
Cellular component biogenesis
Response to stress
Cell division
Proteolysis
Cell septum assembly
Signal transduction
E
Localization
Transmembrane transport
Ion transport
Respons to stimulus
Cellular component biogenesis
Structural constituent of ribosome
Translation
Gene expression
Regulation of nucleic-acid-templated transcription
F
P-site
MFE
OUP-containing genes
Non-OUP genes
Efficiently translated
Inefficiently translated
G
GUG 6%
UUG 3%
AUG 91%
OUP
total = 355
UUG 12%
GUG 9%
AUG 79%
non-OUP
total = 1800

Figure S20.
A. Nucleotide sequence logos generated from all *Listeria* genes containing an OUP of at least ten reads (left) or all other translated genes (right).
B. Box and whisker plots showing distance in nucleotides between the SD sequence and the start codon for efficiently translated genes (top 100 genes by translation efficiency, green), inefficiently translated genes (bottom 100 genes by translation efficiency, red) and all genes (total, grey) in *Salmonella* and *Listeria*.
C. Histogram displaying the number of *Listeria* OUP (blue) or non-OUP (grey) genes that contain a specified distance between the SD and start codon.
D. Bar chart showing the enrichment strength of and the number of genes that belong to a gene ontology group among *Listeria* OUP genes. These groups can overlap.
E. Euler diagram showing the overlap of selected gene ontology groups in *Listeria* OUP genes.
F. Sliding-window average MFE of predicted secondary structure at each nucleotide of sequences surrounding the start codon of OUP containing genes that contain at least 10 reads of the OUP (blue), genes not containing an OUP (black), efficiently translated genes (green, top 100 genes by TE) and inefficiently translated genes (red, bottom 100 genes by TE). The OUP footprint (24 nt) is coloured in pale blue whilst the P-site is highlighted in magenta.
G. Pie charts showing the proportion of genes that start with a specific start codon among OUP genes (left) or non-OUP genes (right).

Figure S21

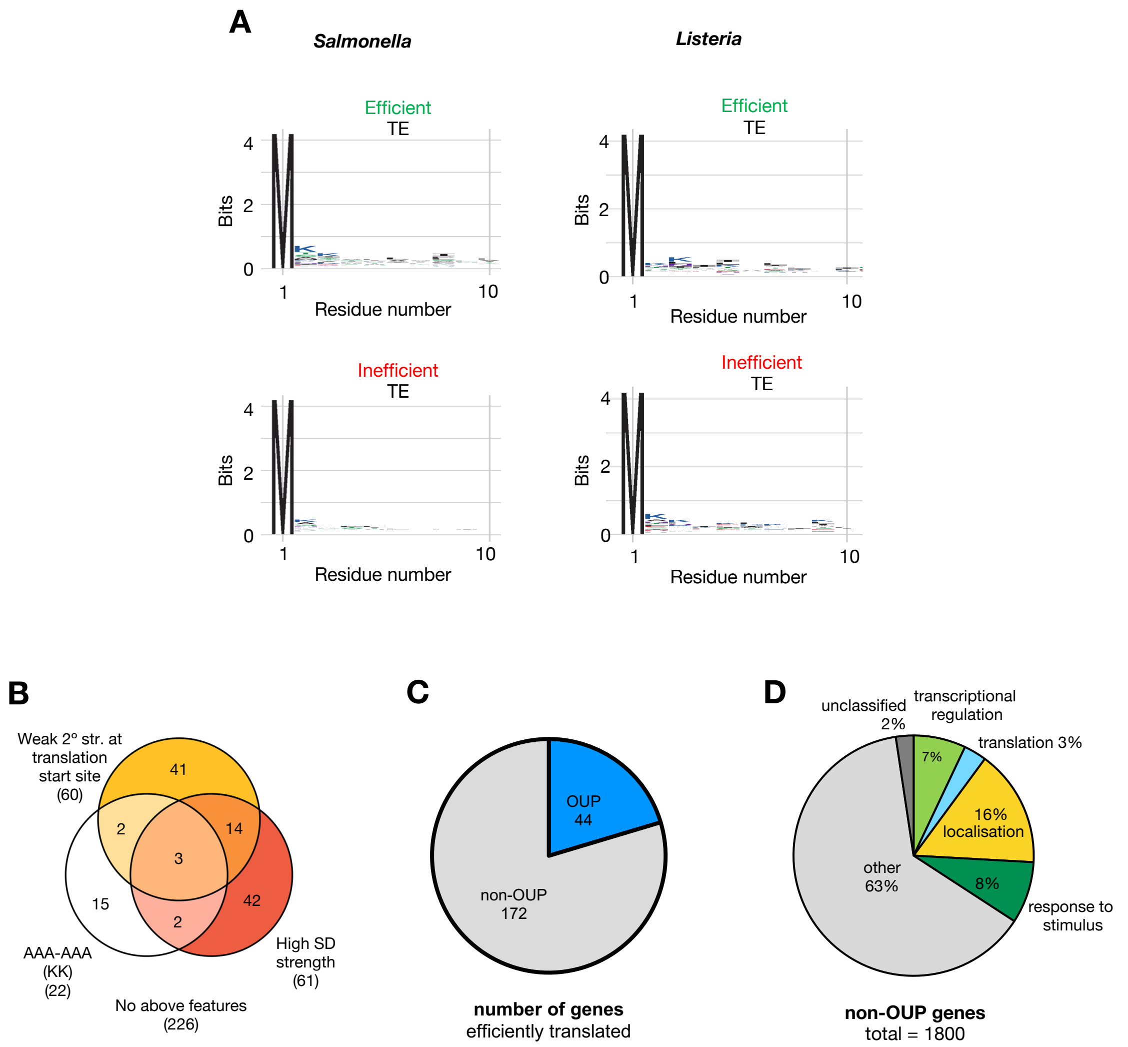
A
Salmonella
Listeria
Efficient
TE
Inefficient
TE
Bits
Residue number
B
Weak 2° str. at translation start site (60)
41
2
14
3
15
42
2
AAA-AAA (KK) (22)
High SD strength (61)
No above features (226)
C
OUP 44
non-OUP 172
number of genes efficiently translated
D
unclassified 2%
transcriptional regulation
7%
translation 3%
16% localisation
other 63%
8%
response to stimulus
non-OUP genes
total = 1800

Figure S21.
A. Sequence logos of N-terminal amino acid sequences of the top 100 most efficiently translated genes (top) or the top 100 least efficiently translated genes (bottom) in *Salmonella* (left) and *Listeria* (right).
B. Venn diagram of efficiently translated genes (top 10% by TE) that contain the following features: weak secondary structure at the translation start site (bottom 10% by average MFE), strong Shine–Dalgarno sequence (top 10% by SD strength), or AAA-AAA codons immediately following the start codon.
C. Pie chart showing the proportion of efficiently translated *Listeria* genes (top 10% by TE) that either contain an OUP or do not.
D. Pie chart of gene ontology groups of non-OUP-genes. The ‘response to stimulus’ group excludes genes that also belong to ‘transcriptional regulation’, ‘translation’ and localisation’.

Figure S22

A

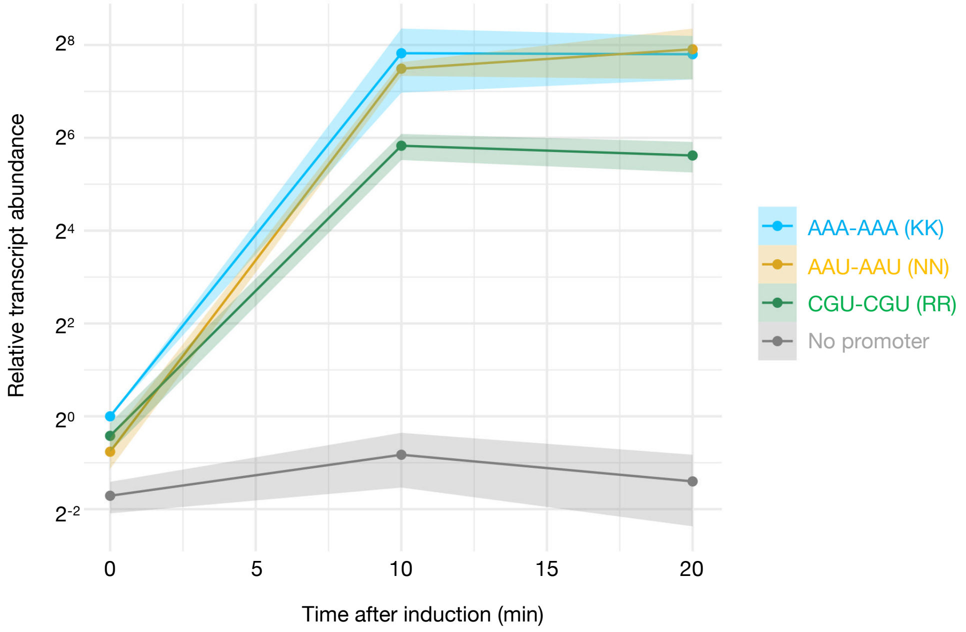

B

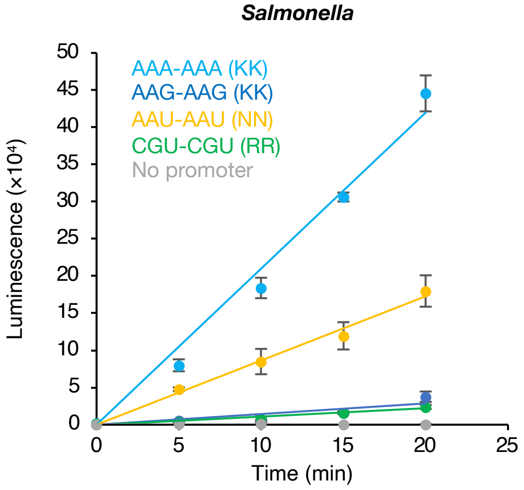

Figure S22.
A. Transcript levels from induction time courses of luciferase expression from constructs containing N-terminal fusions of methionine-lysine-lysine (MKK, blue), methionine-asparagine-asparagine (MNN, yellow), methionine-arginine-arginine (MRR, green), or a construct lacking a promoter (P-less, grey) in *Salmonella* SL1344. The relative transcript abundance was determined by RT-qPCR with *lppA* (SL1344_1311) as a reference housekeeping gene and MKK at time point 0 as the reference sample. Three biological replicates were performed. The ribbons represent the standard error of the mean relative transcript abundance.
B. Induction time courses of LuxA luciferase expression measured by luminescence. Constructs containing AAG-AAG (KK, dark blue), AAA-AAA (KK, light blue), AAU-AAU (NN, orange) or CGU-CGU (RR, green) or a construct lacking a promoter (grey) were expressed in *Salmonella* (SL1344).

Table S1 Sequencing depth of ribosome profiling and RNA-Seq libraries

	filtered genes with mapped RPFs and RNA-Seq reads / total of annotated genes	number of phased RPFs mapping to CDS	number of mapped phased RPFs per kilobase of all CDS	number of RNA-Seq reads mapping to CDS	number of mapped RNA-Seq reads per kilobase of all CDS
Salmonella	3450/4682	19455274	4452	15936291	3601
Listeria	2155/2800	76297470	29577	57690643	22247

Table S2 New open reading frames in *Salmonella* and *Listeria* identified by ribosome profiling

bacterium	candidate	start	end	length	note
Listeria 10403S	L1	733795	733739	57	Upstream of LMRG_00410, which encodes a fibronectin-binding protein.
Listeria 10403S	L2	1235965	1235858	108	Downstream of and in frame with LMRG_00701, which encodes an uncharacterised protein.
Listeria 10403S	L3	1660893	1660795	99	Between LMRG_01314 (cell surface protein gene) and LMRG_01315 (ABC transporter gene).
Listeria 10403S	L4	2320613	2320506	108	Downstream of LMRG_01557, which encodes a hypothetical protein.
Listeria 10403S	L5	2595223	2595071	153	Located between *argS* (Arginine–tRNA ligase) and *rpoE* (Probable DNA-directed RNA polymerase subunit delta).
Listeria 10403S	L6	2723079	2722969	111	Downstream of LMRG_02232, which encodes a DUF5626 family protein.
Listeria 10403S	L7	2747155	2747060	96	Downstream of LMRG_01984, a glucokinase-encoding gene and upstream of ffs ncRNA.
Salmonella SL1344	S1	974971	975054	84	overlaps *ybjX* antisense, upstream of *ybjY*
Salmonella SL1344	S2	4837208	4837369	162	upstream of *yjjU*
Salmonella SL1344	S3	3271980	3271831	150	overlaps antisense *yqgC*. *yqgC* is not annotated in SL1344, it is annotated in LT2, but does not seem to be expressed.
Salmonella SL1344	S4	3594506	3594384	123	overlaps antisense with *yrdA*

Table S3 Copy number of proteins identified from the literature and corresponding RNA-Seq, Ribo-Seq and TE values

gene	Reported copy number	strain	medium	Growth temperature (ºC)	Growth stage at harvest	RNA-Seq	Ribo-Seq	Translation efficiency
argS	4141.6	NC3	RDM	37	exponential	485.699367	172.262364	2.81953268
clpA	270	X90	RDM	37	exponential	392.846511	415.46785	0.94555213
clpP	1400	X90	RDM	37	exponential	404.508325	802.011338	0.50436734
clpX	450	X90	RDM	37	exponential	556.299751	735.999355	0.75584272
dacA	790	MC4100	LB	35	late exponential	281.300154	202.061151	1.39215357
dacB	120	MC4100	LB	35	late exponential	80.5606696	40.3267571	0.50057624
dacC	221	MC4100	LB	35	late exponential	93.8546619	88.0359713	0.93800318
dnaA	1200	FH1218	LB+glucose	unknown	exponential	108.334644	95.4775398	0.881320477
dnaG	75	HMS83	special	unknown	fermenter	361.348352	20.3569083	0.056335966
dps	6000	W3110	LB	37	early stationary	152.978374	343.398842	2.244754157
envZ	100	MC4100	LB	37	exponential	103.639642	13.0777539	0.126184862
fis	60000	W3110	LB	37	early stationary	1339.9039	578.9162	0.432057999
ftsA	200	D1210	LB+glucose	37	exponential	511.715081	524.402419	1.024793755
ftsI	132	MC4100	LB	35	late exponential	170.201105	36.1427283	0.212353076
fusA	59160	NC3	RDM	37	exponential	5411.52	8173.51136	1.510391046
glnS	4296.8	NC3	RDM	37	exponential	193.159888	151.574273	0.78470885
gltX	5526.4	NC3	RDM	37	exponential	339.816243	245.58278	0.722692883
glyS	6241.6	NC3	RDM	37	exponential	329.918809	620.534368	1.880869932
hfq	55000	W3110	LB	37	early stationary	929.496328	1576.47958	1.696057885
hns	20000	W3110	LB	37	early stationary	7597.22851	21853.8996	2.876562106
ileS	4605.6	NC3	RDM	37	exponential	382.77238	497.087309	1.29864989

infA	20400	MRE600	supplemented MOPS	37	exponential	1043.35609	1829.79291	1.753756867
infB	22440	MRE600	supplemented MOPS	37	exponential	431.030011	360.066325	0.835362541
infC	15708	MRE600	supplemented MOPS	37	exponential	3922.38321	821.870365	0.209533419
lepB	820	MC4100	unknown	unknown	unknown	234.157444	186.346643	0.795817721
leuS	4060	NC3	RDM	37	exponential	216.260173	467.534696	2.16190845
lexA	1300	AB1157	EYM9	37	exponential	991.75681	700.019042	0.705837394
lppA	500000	unknown	unknown	unknown	unknown	35405.0616	48752.1591	1.376982752
lysS	4416	NC3	RDM	37	exponential	1223.53067	1556.16154	1.271861489
mrcA	221	MC4100	LB	35	late exponential	69.8686435	19.4175376	0.277914907
mrcB	127	MC4100	LB	35	late exponential	148.723588	85.650509	0.575903999
mrdA	120	MC4100	LB	35	late exponential	43.5569864	17.8735879	0.410349507
mscL	480	MG1655	LB	37	late exponential	501.815759	780.029146	1.554413411
pheS	5502	NC3	RDM	37	exponential	136.407365	148.835706	1.091111955
pheT	4224	NC3	RDM	37	exponential	660.544931	293.706577	0.444642845
prfA	4900	MG1655	phosphate buffered	37	stationary	60.7608517	61.3037341	1.00893474
prfB	24900	MG1655	phosphate buffered	37	stationary	1119.58721	194.986991	0.174159717
recA	7200	AB1157	EYM9	37	exponential	996.385638	1130.72758	1.134829264
rpoA	22809.6	NC3	RDM	37	exponential	9555.56713	2030.89639	0.212535411
rpoB	13000	MG1655	M9 complete	30	exponential	885.223173	1336.50118	1.509790093
rpoD	17000	MG1655	M9 complete	30	exponential	914.312878	773.352244	0.845828887
rpoE	5500	MG1655	M9 complete	30	exponential	288.433253	38.0630237	0.131964755
rpoH	120	MG1655	M9 complete	30	exponential	1170.1326	390.006703	0.333301288
rpsA	71131.2	NC3	RDM	37	exponential	7103.79914	5813.84591	0.818413611
ssb	8000	C600	LB+thiamine	unknown	exponential	1182.39285	1153.65495	0.975695139

thrS	3874.4	NC3	RDM	37	exponential	657.411696	389.613821	0.592648143
tsf	43675.2	NC3	RDM	37	exponential	4240.04514	4297.24789	1.013491069
valS	3526.4	NC3	RDM	37	exponential	297.270891	408.899605	1.375511756

Table S4 Reagents used for Ribo-Seq and RNA-Seq library generation

Profiling buffer	20 mM Tris-HCL,
	140 mM KCl,
	5 mM MgCl2,
	0.5% w/v NP-40,
	1% v/v Triton X-100
	35% PGB
	0.5 mM DTT,
	1500 µg/ml chloramphenicol
	100 U/ml DNase I
2x fragmentation buffer	2 mM EDTA pH 8.0,
	12 mM Na2CO3,
	90 mM NaHCO3
Alkaline hydrolysis stop solution	0.3 M NaCH3COO

Table S5 5'-P-site distances for ribosome protected fragments of specified length in *Salmonella* and *Listeria* Ribo-Seq libraries

Read length	23	24	25	26	27	28	29	30
Salmonella SL1344	8 (2)	8 (2)	9 (1)	10 (3)	11 (2)	12 (1)	13 (3)	14 (2)
Salmonella SJW1103	8 (2)	8 (2)	9 (1)	10 (3)	11 (2)	12 (1)	13 (3)	14 (2)
Listeria 10403S	11 (2)	11 (2)	12 (1)					

5′ position in codons in brackets.

Supplementary methods

Obtaining highly phased RiboSeq data in bacteria presents multiple challenges, which we have overcome by: (1) adding a large excess of chloramphenicol to cultures to prevent incomplete translation arrest which can 'blur' ribosome positions, followed by rapid cooling and flash freezing in liquid nitrogen; (2) use of RNase I instead of nucleases with significant sequence specificity such as S7 MNase, and (3) combining two different rRNA subtraction methods (RiboCop and DSN treatments) to significantly enrich for mRNA reads in our RiboSeq libraries without introducing significant biases. Together, the protocol described in this manuscript allowed us to obtain data with features typically seen in eukaryotic ribosome profiles where reads have a strongly peaked size distribution, specific to the coding sequence and show strong triplet phasing, therefore enabling accurate analysis of translational features. The ability to obtain highly phased RiboSeq data in *Salmonella* and *Listeria* indicates that *Salmonella* and *Listeria* – robust models for pathogenicity of Gram-negative and Gram-positive bacteria – are also powerful systems to study global bacterial translation.

To prepare cells for RiboSeq and parallel RNASeq, we grew cells to exponential phase prior to arresting translation and capturing stalled ribosomes (Fig. S1). Translation can be arrested with antibiotics that target the bacterial ribosome, inhibiting elongation and trapping assembled ribosomes on mRNAs[1,2]. Several RiboSeq studies have shown that translation inhibitors such as cycloheximide or chloramphenicol can artificially result in ribosome accumulation at the 5′ end of ORFs due to incomplete or heterogenous inhibition of elongating ribosomes[3,4]. Other strategies of harvesting bacterial cells and arresting translation include filtering

cultures or rapid freezing, which can also introduce artefacts[4]. One approach developed to prevent incomplete translation arrest in eukaryotic RiboSeq studies is to use a large excess of the eukaryotic translation inhibitor, cycloheximide[3]. To circumvent incomplete translation arrest in our data, we arrested translation by treating cells with an excess of highly concentrated chloramphenicol, followed by rapid cooling and flash freezing cells in liquid nitrogen immediately prior to cryo-lysis (as detailed in Materials and Methods). As shown later, we did not observe an increase in ribosome footprint density at the 5′ end of ORFs, indicating that artefacts due to chloramphenicol treatment did not arise under our modified conditions (Fig. 1A, 1C, 1D, 2A, 2C, S5). Nevertheless, for downstream analysis, we took a further precaution to eliminate any potential artefacts near initiation sites by excluding reads with 5′ ends mapping outside of the coding region prior to further analysis.

Artefacts in bacterial RiboSeq studies can also arise due to the choice of nuclease used to generate RPFs[5,6]. Eukaryotic RiboSeq libraries are typically generated using RNase I nuclease, whereas *E. coli* ribosomes reportedly inhibit RNase I, meaning alternative nucleases such as S7 MNase that exhibit higher sequence specificity have previously been used to generate RPFs. The higher sequence specificity of S7 MNase however results in libraries with high levels of noise, resulting in libraries that lack phasing. We generated RiboSeq libraries from *Salmonella* using our modified method and used both S7 MNase and RNase I nucleases. We found that treatment with S7 MNase resulted in data without phasing, consistent with previous reports from bacterial RiboSeq libraries generated with S7 MNase[4–7] (Fig. S2B). Treatment with RNase I, however, resulted in RPFs with a distinct size distribution that are phased, indicating RNase I can be used to generate *Salmonella* RiboSeq data with

single nucleotide resolution visible at the individual gene level (Fig. 1A, 1C, 1D, 2A, 2C, S5). To confirm reproducibility of our protocol, we applied the afore mentioned method on the pathogenic *Salmonella* strain SL1344, other LT2 derived *Salmonella* strain as well as *Listeria* 10403S to generate highly-phased RiboSeq libraries.

Supplementary references

1. Wilson, D. N. Ribosome-targeting antibiotics and mechanisms of bacterial resistance. *Nat. Rev. Microbiol.* **12**, 35–48 (2014).
2. Lin, J., Zhou, D., Steitz, T. A., Polikanov, Y. S. & Gagnon, M. G. Ribosome-Targeting Antibiotics: Modes of Action, Mechanisms of Resistance, and Implications for Drug Design. *Annu. Rev. Biochem.* **87**, 451–478 (2018).
3. Gerashchenko, M. V. & Gladyshev, V. N. Translation inhibitors cause abnormalities in ribosome profiling experiments. *Nucleic Acids Res.* **42**, e134–e134 (2014).
4. Mohammad, F., Green, R. & Buskirk, A. R. A systematically-revised ribosome profiling method for bacteria reveals pauses at single-codon resolution. *Elife* **8**, (2019).
5. Oh, E. *et al.* Selective Ribosome Profiling Reveals the Cotranslational Chaperone Action of Trigger Factor In Vivo. *Cell* **147**, 1295–1308 (2011).
6. Li, G.-W., Oh, E. & Weissman, J. S. The anti-Shine–Dalgarno sequence drives translational pausing and codon choice in bacteria. *Nature* **484**, 538–541 (2012).
7. Li, G.-W., Burkhardt, D., Gross, C. & Weissman, J. S. Quantifying Absolute Protein Synthesis Rates Reveals Principles Underlying Allocation of Cellular

Resources. *Cell* **157**, 624–635 (2014).
